# Application of ChatGPT in pediatric surgery and further issues that should be addressed: comment

**DOI:** 10.1097/JS9.0000000000001459

**Published:** 2024-04-09

**Authors:** Hinpetch Daungsupawong, Viroj Wiwanitkit

**Affiliations:** a Private Academic and Editorial Consultant, Phonhong, Laos; b University Centre for Research & Development Department of Pharmaceutical Sciences, Chandigarh University Gharuan, Mohali, Punjab, India

*Dear Editor,*

We would like to discuss ‘Application of ChatGPT in pediatric surgery: opportunities and challenges’^1^. Mao *et al*. raise many interesting concerns about using ChatGPT in clinical surgery. Nevertheless, some specific issues should be further addressed. One critical factor to consider while utilizing ChatGPT in pediatric surgery is incorporating the child’s parents or guardians in decision-making. Children cannot provide informed permission for their treatment since they are minors, so it is critical to ensure that their caregivers are well-informed and engaged in the treatment plan. This involves explaining ChatGPT’s recommendations and soliciting feedback before taking any action. Furthermore, healthcare providers should be aware of the possibility of biases or mistakes in the data used to train the AI (artificial intelligence) model, especially for pediatric patients who may have specific healthcare demands or diseases. Furthermore, precise rules and protocols for the use of AI technology in pediatric surgery are required to assure patient safety and confidentiality at all times. This includes implementing strong data protection measures, securing patient information, and continually upgrading the AI model with the most recent evidence-based procedures in pediatric surgery. By addressing these legal and ethical concerns, healthcare professionals can leverage AI technologies like ChatGPT to improve decision-making processes and patient outcomes in pediatric surgery.

Here, we also would like to add additional issues that should be addressed. The two issues that are hereby mentioned are legal and ethical issues. The capacity of ChatGPT to make suggestions is the main focus of many studies, although this may not adequately convey the complexity of clinical decision-making while treating malignant otitis externa. The recommendations made by ChatGPT are predicated on data collected before April 2003, which could not include the most recent findings or newly developed therapies that haven’t been well covered by the media. The study evaluates ChatGPT’s usefulness in producing new possibilities; however, it does not include a thorough validation procedure to verify the effectiveness and security of these AI-generated suggestions in clinical settings. Relying solely on a panel of experts to make assessments is subjective and may not fully account for patient-specific characteristics or represent the larger medical consensus. Additionally, the study’s statistical examination of inter-observer variances is inadequate. As previously indicated, numerous recently published articles on applied AI in reputable journals have the potential to both support ChatGPT’s recommendations and cast doubt on the value of physicians and otolaryngologists. Because the publications are open access, people may abuse their contents to treat themselves using ChatGPT recommendations and to obtain legal advice regarding the prescription regimens and treatment plans prescribed by medical professionals. And last, the moral implications of AI use are a significant subject. Norms or standards can be created, and any ethical conundrums can be investigated to ensure that AI-generated content is used in an ethical and transparent manner^2^.

## Ethical approval

Not applicable.

## Consent

Not applicable.

## Sources of funding

The author declares no conflict of interest.

## Author contribution

H.D.: 50% ideas, writing, analyzing, and approval; V.W.: 50% ideas, supervision, and approval.

## Conflicts of interest disclosure

The author declares no conflict of interest.

## Research registration unique identifying number (UIN)

Not applicable.

## Guarantor

Professor Viroj Wiwanitkit.

## Data availability statement

There are no new data generated.

## Provenance and peer review

Not commissioned, externally peer-reviewed.
